# Evaluation of Welfare in Commercial Turkey Flocks of Both Sexes Using the Transect Walk Method

**DOI:** 10.3390/ani11113253

**Published:** 2021-11-13

**Authors:** Nina Mlakar Hrženjak, Hristo Hristov, Alenka Dovč, Jana Bergoč Martinjak, Manja Zupan Šemrov, Zoran Žlabravec, Jožko Račnik, Uroš Krapež, Brigita Slavec, Olga Zorman Rojs

**Affiliations:** 1 Institute of Poultry, Birds, Small Mammals and Reptiles, Faculty of Veterinary Medicine, University of Ljubljana, Gerbičeva Ulica 60, 1000 Ljubljana, Slovenia; nina.mlakarhrzenjak@vf.uni-lj.si (N.M.H.); alenka.dovc@vf.uni-lj.si (A.D.); zoran.zlabravec@vf.uni-lj.si (Z.Ž.); josko.racnik@vf.uni-lj.si (J.R.); uros.krapez@vf.uni-lj.si (U.K.); brigita.slavec@vf.uni-lj.si (B.S.); 2 Nutrition Institute, Tržaška Cesta 40, 1000 Ljubljana, Slovenia; hristovhristo@outlook.com; 3 Perutnina Ptuj, Perutnina Ptuj d.o.o., Potrčeva 10, 2250 Ptuj, Slovenia; Jana.Brgoc@perutnina.eu; 4 Department of Animal Science, Biotechnical Faculty, University of Ljubljana, Groblje 3, 1230 Domžale, Slovenia; manja.zupansemrov@bf.uni-lj.si

**Keywords:** welfare, mixed commercial turkey flocks, on-farm assessment

## Abstract

**Simple Summary:**

In the last decade, increased attention has been directed toward the welfare of commercial poultry. In current turkey production systems, males and females are typically reared in the same facility until slaughtering the hens. Hens are reared for 12 to 14 weeks, while toms are reared for up to 22 weeks. This study examines farm health and welfare in commercial turkey flocks of both sexes during the fattening cycle using the transect walk method. Flocks, separately for males and females, were assessed at 3 to 4 weeks of age, 1 week before slaughtering the hens and 1 week before slaughtering the toms. We found several differences in the frequency of welfare indicators between different assessments and between male and female populations. The period just before slaughtering the hens was found to be most problematic for both sexes, although several welfare indicators suggested that health problems were mainly already present at 3 to 4 weeks of age and also continued after hen depopulation. Our results show that transect walks used at different ages may provide relevant information on animal health and welfare during the fattening cycle.

**Abstract:**

The study was conducted between March and September 2019 in six meat-type turkey flocks with similar management standard procedures using the transect walk method. The concept of the method is based on visual observation of the birds while slowly walking across the entire farm in predetermined transects. Each flock was evaluated at three different times during the fattening cycle: at 3 to 4, 12 to 13, and 19 to 20 weeks of age, and total number of males and females that were immobile or lame, had visible head, vent, or back wounds, were small, featherless, dirty, or sick, had pendulous crop, or showed aggression toward birds or humans were recorded. At each visit, NH_3_ and CO_2_ were measured within the facilities. In the first assessment, the most frequently observed welfare indicators were small size (0.87%) and immobility (0.08%). Males showed a significantly higher prevalence of small size (*p* < 0.01), sickness (*p* < 0.05), and dirtiness (*p* < 0.1) compared to females. In the second assessment, the most common findings in both sexes were dirtiness (1.65%) and poor feather condition (1.06%), followed by immobility (0.28%). Males were significantly dirtier (*p* < 0.001), had more immobile birds (*p* < 0.01) and birds with vent wounds (*p* < 0.1), but had fewer sick birds (*p* < 0.05). In the last assessment, an increase in immobile, lame, sick, and dead birds was recorded, indicating an increase in health problems. Higher CO_2_ (3000 and 4433 ppm) and NH_3_ (40 and 27.6 ppm) values were noted only at the first assessment in two facilities. Further analyses showed that slightly elevated NH_3_ and CO_2_ levels did not influence the occurrence of welfare indicators. This study is the first description of the welfare of commercial turkey flocks in Slovenia.

## 1. Introduction

In the last decade, increased attention has been directed toward the welfare of commercial poultry and its assessment. Not only the awareness of the public, but also of farmers and stakeholders, is increasing due to the obvious impact of animal welfare issues on production [1,2,3,4].

Several on-farm animal-based assessment methods have been introduced to evaluate the welfare of poultry. In 2009, the Welfare Quality Assessment Protocol was introduced for layers and broilers [5], but it proved to be complex and time consuming [6], and it could even pose a risk to birds or handlers when used for other poultry species because it requires handling the birds. On the other hand, the transect walk method has proven to be practical, reliable, and efficient without the need to catch birds or subject the flock to excessive stress. The method has been used successfully in large flocks of broilers [1,2,3]. and in turkeys [7,8,9]. The concept of the method is based on visual observation of the birds while slowly walking across the entire farm in predetermined transects, as is usually done during routine inspection by farmers. While walking, the observer records every bird affected by health and welfare indicators such as small size, dirty or featherless birds, birds with head, back, or tail wounds, immobile, lame, sick, terminally ill, or dead birds, and birds showing aggressive behavior toward other birds or humans [10]. The transect walk can be used at different ages in turkeys, provides relevant information on animal welfare during the rearing period, and allows farmers to make changes and improve welfare for current flocks [11]. The health and welfare of commercially farmed turkeys supposed to be attributed to the high growth potential of the commonly used commercial hybrids [12,13], and also depend on environmental factors such as air quality [14,15], ambient temperature [16,17], light intensity and duration of day length [18,19], and stocking density [2,20]. All these factors, if not within the recommended limits, can cause significant physical distress to the animals [16,17] and consequently have a negative impact on animal performance [2,20], and post-slaughter product quality [21,22,23].

In Slovenia, commercial turkeys are reared in a conventional housing system and, as in many other countries, birds of both sexes are kept in the same facilities separated by the wire mash until slaughtering the hens. Hens are slaughtered when the birds reached an average body weight of 9 kg at around 14 weeks, while toms are reared until 21 to 22 weeks of age. There are limited field studies on welfare in such mixed flocks during the fattening period because most previous studies have been carried out before slaughter [7,8,11]. The aim of this study was to identify transect-based on-farm welfare indicators of commercial turkeys of both sexes at three different points in time during the fattening cycle; 3 to 4 weeks after placement and before slaughtering the hens and toms. We hypothesized that the welfare problems identified would differ at different ages and between males and females. Namely, the first weeks after placement of turkey chicks on farms are critical due to health problems caused by infectious diseases such as colibacillosis and aspergillosis [24,25] and specific behavior and environmental requirements of poults [1]. The second and third time points were chosen because in these two periods the facilities are at their maximum capacity regarding stocking density and ventilation, which may influence animal welfare [9,26]. In addition, we investigated the importance of selected climate conditions on the occurrence of animal welfare indicators.

## 2. Materials and Methods

### 2.1. Meat-Type Turkey Flocks

The study was conducted between March and September 2019 on six meat-type turkey flocks of both sexes (hens and toms) of two hybrids (Converter and British United Turkeys (B.U.T.) Big 6). All flocks were kept on farms in the central region of Slovenia. The owners of the farms were subcontractors of one poultry producer; therefore, it was expected that the management practices would be similar. The size of the houses varied between 900 and 1440 m^2^, and the number of birds housed ranged from 4300 to 7300. All birds were beak trimmed in the hatchery. Males and females were housed separately, but in the same house, with the toms occupying about 60% of the area until the hens were slaughtered, after which the toms were placed throughout the entire area. From flocks 1 and 2, about one-third of the animals were removed at the age of 38 days and placed on another farm. Birds from all flocks were vaccinated against Newcastle disease and hemorrhagic enteritis. The number of birds found dead or that were culled was recorded daily. When health problems were noticed by the poultry farmers, a field veterinarian inspected the flock. Based on the results of the clinical observations and pathological findings, a decision on treatment was made where appropriate. The flock’s information is summarized in Table 1.

All facilities were fully enclosed and insulated. They had a concrete floor and were equipped with either manually or automatically controlled ventilation systems, automatic drinkers, and automatic feeders. The birds were reared on wood shavings. The natural light entering the house through the windows was supplemented by artificial lighting for a total of 23 to 24 h of light per day during the whole rearing period. The light intensity varied from 3 to 27 lux. Birds did not have any environmental enrichment and access to elevated areas. All flocks had the same feed supplier and were slaughtered in the same slaughterhouse. The hens were slaughtered at around 14 to 15 weeks of age, when the birds reached an average body weight (BW) of 9 kg. Toms were slaughtered when the animals reached an average BW of 20 kg at 21 to 22 weeks of age. The flocks’ information is summarized in Table 1.

### 2.2. Evaluation of Animal Welfare

Each flock was visited at three different times during the fattening cycle; the first visit took place at 3 to 4 weeks of age, the second visit took place approximately 1 week before slaughtering the hens (i.e., at 12 to 13 weeks of age), and the last assessment was conducted before slaughtering the toms at 19 to 20 weeks of age (Table 1). All visits were carried out by the same observers. At each assessment, information on cumulative mortality was collected from farm records, and stocking density was calculated for each flock, for males and females separately (Table 2).

The transect walk approach methodology developed by Marchewka et al. (2015) [7] was used to assess the welfare of commercial turkeys. At the first and second visits, toms and hens were assessed on the same day. Because both male and female animals were housed in the same house, each part (male and female) was divided into three to four longitudinal transects, depending on the size of the building. This approach was used in the first two evaluations. In the last assessment only toms were present, and therefore the entire barn was divided into three to four longitudinal sections. The assessor walked through the transect parts, from the entrance wall to the wire mesh or to the opposite wall (third assessment). The observer moved slowly to minimize disturbance to the birds during the assessment and recorded all observed occurrences of birds that fell into any of the predefined animal welfare indicator categories shown in Table 3.

### 2.3. Environmental Parameters

Inside temperature, ammonia (NH_3_), and CO_2_, were checked using Dräger X-am 1/2/5000 (Dräger, Lübeck, Germany) in each facility. All measurements were performed at animal level at six different locations: left and right at the entrance, in the middle, and at the end of the facility. Average values were calculated for each parameter.

### 2.4. Statistical Analyses

Incidence of welfare indicators were calculated for each flock, for males and females separately, and therefore the analyses were conducted with the flock and sex as experimental unit. Data were analyzed using STATA version 15.1 (StataCorp LLC, Lakeway Dr, College Station, TX, USA). A *Z*-test for difference in proportions was used to analyze differences between male and female populations in the occurrence of different welfare indicators. Logistic regression analyses were used to determine the effect of CO_2_ and NH_3_ on animal welfare represented by the presence of indicators that describe the level of animal welfare, including mortality.

## 3. Results

### 3.1. Evaluation of Animal Welfare

**Figure 1:**
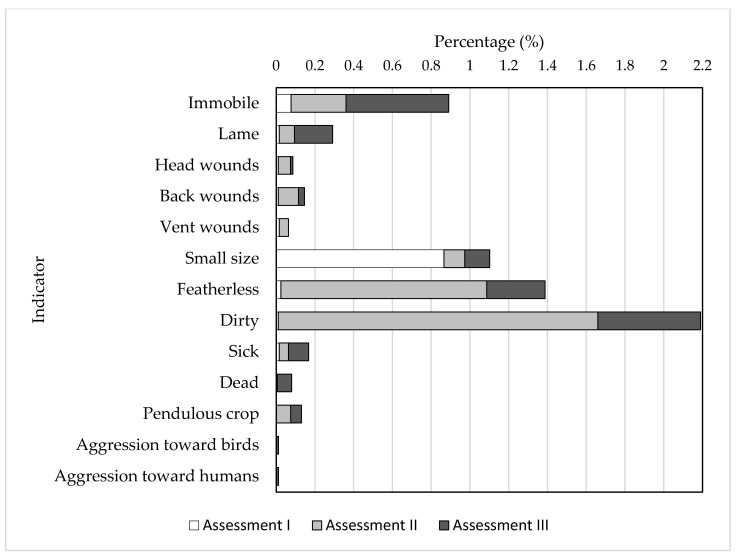
Mean values of welfare indicators recorded in meat-type turkeys at 3 to 4 (Assessment I), 13 to 14 (Assessment II), and 17 to 19 weeks of age (Assessment III).

The mean values of each welfare indicator recorded at each assessment in male and female turkeys are presented in Table 4 and Figure 1.

In the first assessment, the most frequently observed welfare indicators in both males and females were small size and immobility. Overall, 0.997% of the males and 0.721% of the females were half the size of the other birds. Immobility was observed in 0.076% of male and 0.078% of female birds. All other indicators were rarely or never observed. No birds with pendulous crop or aggressive behavior were noted at this age. There were significantly smaller (*p* < 0.01), sick (*p* < 0.05), and dirty birds (*p* < 0.1) among males compared to females.

In the second assessment, the most common findings in both sexes were dirtiness and poor feather condition, followed by immobility. Males were significantly dirtier (*p* < 0.001), and there were more immobile birds (*p* < 0.002) and birds with vent wounds (*p* < 0.100), but fewer sick birds (*p* < 0.048) compared to females. At this assessment, pendulous crop was observed in both sexes, but the difference was not significant. No aggressive behavior was found.

The most common welfare indicators found in males before slaughter were immobility and dirtiness (0.53%), followed by poor feather condition (0.302%), lameness (0.197%), small size (0.129%), and sick birds (0.105%). At this age, aggression toward humans and other birds was also observed, but in very few birds (0.012%).

### 3.2. Effects of Selected Environmental Parameters on the Presence of Welfare Indicators

The average CO_2_ and NH_3_ values and temperature within each turkey facility obtained at each assessment are presented in Table 5.

The average CO_2_ values ranged from 850 to 4433 ppm. The values above the recommended (CO_2_ < 2500 ppm) were recorded at the first assessment in flocks 1 and 2. At that time point, the NH_3_ level in both facilities exceeded the anticipated level of 20 ppm. At the second and the third assessment, NH_3_ values were low in all flocks and did not exceed 4 ppm. The CO_2_ values were also low and ranged from 850 to 2200 ppm. Inside temperatures recorded at all three visits were slightly higher than recommended for the specific age of turkeys [27].

Table 6 shows the influence of different CO_2_ and NH_3_ levels on occurrences of welfare indicators and mortality.

For NH_3_, the probability of occurrence of welfare indicators was lower at concentrations of NH_3_ > 0 ppm compared to NH_3_ = 0 ppm; for CO_2,_ the possibility of the development of welfare indicators was higher at CO_2_ < 1600 ppm compared to CO_2_ between 1600 and 3000 ppm and more than 3000 ppm.

## 4. Discussion

In Slovenia, turkeys represent less than 2% of poultry meat production and, until this study, no data were available on the welfare profile of this species. The aim of this study was to identify transect based on-farm health and welfare indicators in commercial turkey flocks of both sexes during the fattening cycle. Flocks, separately for males and females, were assessed at 3 to 4 weeks of age, 1 week before slaughtering the hens, and 1 week before slaughtering the toms. In brief, we found several differences in the frequency of welfare indicators between different assessments and between male and female populations.

To date, no data on the welfare of turkeys based on the transect walk approach at the age of 3 to 4 weeks are available. The results of our study showed that small birds were identified as the main welfare problem at this age. Such birds were found in both sexes, although significantly more small birds were observed in the male population. The second most common indicator was immobility. A high incidence of smaller birds and immobility may indicate compromised health and welfare on a farm due to either general housing or bird health problems. Infections caused by *E. coli* are commonly present in young chicks. Localized infections such as omphalitis and yolk sac infection or systemic colibacillosis generally resulted in higher mortality in the first weeks after placement. Affected birds are usually undersized, because they may have difficulties in walking, which alters weight and leads to weakness [25]. Of the flocks included in the study, colibacillosis was diagnosed in one flock and the birds were treated with antibiotics. Although no veterinary intervention was required in the other flocks, the cumulative mortality indicated that health problems due to *E. coli* or other unidentified infections were present in at least one other flock.

Excessive NH_3_ and CO_2_ levels may also have negative impact on birds’ health and metabolism in young turkeys [14,28]. It was shown that young poults exposed to 4000 ppm CO_2_ had suppressed body weight gain compared to those exposed to 2000 ppm [14]; in addition, NH_3_ levels greater than 10 ppm can also reduce feed intake with effects on body weight [28]. In our study, higher NH_3_ (40 ppm and 27.6 ppm) and CO_2_ (4433 ppm and 3000 ppm) values were detected in two facilities. Elevated levels of both gasses directly correlate with reduced ventilation. Under field conditions, such a situation is often seen in the first weeks after placement of turkeys due to reduced heating costs [29]. Nevertheless, the higher mortality as well as the significantly higher incidence of sick birds found in males indicate that health problems seem to play a more important role than environmental conditions, which were equal for males and females.

As shown in other studies [9], the most problematic period for both sexes in mixed commercial turkey flocks seems to be the time before slaughtering the hens. Indeed, more indicators were present at the second assessment than the first, and the overall prevalence of altered birds was higher compared to the first and third assessments. The most common findings were dirtiness and poor feather condition, followed by immobility. Dirty feathers were observed in more than 2% of males and in 1.204% of females. Dirtiness before slaughtering the hens has also been reported by other authors, although not with such a high prevalence as in our study. In a study conducted in Norway, an average of 0.36% dirty males and 0.15% females were observed at 11 weeks [9,11,30]. In Italian commercial turkey flocks, dirtiness was recorded in 0.022% of females. Unfortunately, males of this age were not scored [8], and so no direct comparison was possible. Previous studies have shown that poor litter quality, dust, and high stocking density can significantly impact dirtiness [1]. Unfortunately, litter quality was not scored in our study. CO_2_ and NH_3_ values that reflect inadequate ventilation and poor litter management [31] were low in all facilities, but the measurements were performed only during assessments so no relevant conclusions could be made on the significance of poor litter quality on such a high prevalence of dirtiness. Recently, dirtiness in turkeys was found to be correlated with immobility and lameness more than with poor litter quality [9]. In our study, immobility was the third most frequently observed indicator at this age. Similar to previously reported findings [8,32], this occurred significantly more frequently in males than in females, which could explain why males were significantly dirtier. Dirty feathers have been suggested as an indicator of health problems of the digestive system [33], but it is unlikely that necrotic enteritis diagnosed at 5 weeks of age in five of six flocks contributed to such a high percentage of dirty birds observed 7 weeks later. Necrotic enteritis is an acute disease caused by toxins produced by *Clostridium perfringens.* The course of the disease is usually short, and birds respond very well to antibiotic treatment if it is given immediately after the onset of the disease [34,35].

Featherless condition was the second most frequently observed welfare indicator during this period. Missing or damaged feathers, particularly in the tail region, were observed in 0.986% of males and in 1.145% of females. The etiology is not entirely known, but it is likely that poor plumage is the consequence of feather pecking. In turkeys, mild feather pecking can be a form of social or investigative behavior [36,37]. When pecking becomes more severe, it may result in severely damaged feathers and feather loss, or even cannibalism [38]. The incidence of feather pecking is known to increase with age, although damaging pecking can occur as early as the 1st or 2nd week of age [39]. Under field conditions, high stocking density, inappropriate lighting, feed deficiency, breed, and sex are considered to influence injurious feather pecking [2,8,40]. Such aggressive pecking often results in wounds seen on the head, around the tail, on the wings, and in the back region. In our study, injuries were rarely found and were observed in both sexes, although vent wounds were found significantly more frequently in males. This is consistent with the results from commercial turkey flocks in Norway [9] and Italy [8]. To prevent feather pecking and aggressiveness, beak trimming is still common practice in commercial turkeys [40], but it seems that beak trimming does not play an essential role in preventing poor feather condition. The incidences of featherless birds observed in our study and in Italian commercial flocks [8]—both included beak-trimmed birds—were higher compared to the study performed in non-trimmed commercial flocks in Norway [9].

The percentage of sick birds was low, but the difference between the sexes was significant. For classifying a bird as sick, other studies also included birds with pendulous crop [7,8,9,11,30]. In our study, birds with pendulous crop were recorded separately, and so no direct comparison could be made. The etiology of pendulous crop is yet not fully understood, but hereditary predisposition, dietary influence, increased liquid intake in hot weather, and the effect of lighting period have been suggested. Unfortunately, no treatment is available, and the carcasses of affected birds are usually condemned at processing [41,42,43]. In our study, birds with pendulous crop were observed in both sexes, although at much lower frequencies compared to the study performed by Vermette et al. [32]. In their clinical study, pendulous crop was found to be the second major reason for morbidity and mortality in turkeys, and females were significantly more affected than males. In comparison to the first assessment, the size of birds was more uniform. This supports previous suggestions that the number of small birds decreases with age [30].

At the last visit, only male turkeys were assessed. Compared to the second assessment, the prevalence of immobile as well as lame, sick, and dead birds increased, indicating health problems, most likely caused by poorer leg health. Lameness and immobility as its consequence are important welfare and health issues in commercial turkey flocks, especially in males [8,32]. Due to their heavy weight and longer production cycle, males’ legs are exposed to more stressors, resulting in chronic pain and movement difficulties [23]. In addition to degenerative and development disorders, bacterial and viral infections such as *Staphylococcus aureus*, mycoplasmas, and reovirus are involved in skeletal and joint lesions, causing acute or chronical local inflammations or even systemic septicemia, resulting in higher mortality [44]. Because no further investigations were performed, we do not know the exact causes of immobility. Dirty and featherless animals were frequently observed, although the prevalence was much lower than in the second assessment. This is consistent with recent findings that poor feather condition decreases with lower stocking density [20,26], but may still persist within the flock due to leg problems [9]. The incidence of head, back, and vent wounds also decreased to less than half after depopulation of females. These results are in agreement with previous findings that injurious feather pecking and wounds may be a consequence of behavioral disturbances due to high stocking density [1]. In the EU, specific minimum stocking density requirements have been established for broilers [45], but these do not directly apply to turkeys. In some countries, such as Norway, specific regulations depending on the live weight of turkeys have been adopted [9] or recommendations for turkeys have been published [46]. Unfortunately, no such documents are available in Slovenia.

During the production cycle, NH_3_ and CO_2_ are the prevalent gases in turkey facilities [47]. NH_3_ in high concentrations can have severe adverse health effects, causing lesions of the upper respiratory tract and inflammation of the cornea and conjunctive [15,48]. High concentration of CO_2_ can be harmful to turkeys due to hypoxia [14], which may lead to dilatated cardiomyopathy [49]. Although no precise concentration limits have been established for turkeys, Directive 98/58/EC [50] requires that gas concentrations in turkey facilities be kept within safe limits. For broilers, EU regulation established the maximum NH_3_ concentration inside a poultry barn at 20 ppm, and for CO_2_ the concentration should not exceed 3000 ppm measured at the level of the chickens’ heads. If higher levels are detected, corrective actions must be taken [45]. In Slovenia, most commercial turkey farms are equipped with sensors for temperature and humidity, but gas concentrations are not measured on a daily basis. Apart from higher CO_2_ and NH_3_ values recorded at two facilities in the first assessment, the levels were low in all facilities in the next two assessments. Similar results were also obtained in the study performed in commercial turkey flocks in Poland, where a significant decreasing trend was observed during the production cycle [47]. Although accurate limits should be defined for turkeys, our results indicate that NH_3_ levels of 40 ppm or less did not influence the occurrence of welfare indicators. Moreover, a higher probability could be expected at NH3 = 0 ppm compared to more than 0 ppm. For CO_2_ the probability of occurrence of animal welfare indicators was higher at levels below 1600 ppm than at levels between 1600 and 3000 ppm or above 3000 ppm. The reason for this could be that birds exposed to slightly elevated NH_3_ or CO_2_ concentrations are likely to be less active. Similar results were recently obtained by Candido et al. [14], who found that poults housed at a lower CO_2_ level (2000 ppm) showed reduced movement compared to those exposed to higher CO_2_ concentrations. However, further studies should be performed to obtain a balance between welfare and the optimal production of turkeys.

## 5. Conclusions

Compared to other poultry species, there is a lack of field studies on welfare problems in commercial turkey flocks. In this study, we investigated some aspects of health and welfare in commercial turkey flocks of both sexes in Slovenia. We cannot assume that our limited sample of flocks is representative of the commercial turkey industry in Slovenia, but it provides an estimation of problems that may exist during the production cycle and emphasizing the importance of setting specific standards and regulations regarding levels of harmful gases and stocking density for commercial turkeys. Our study confirmed that assessing welfare using transect walk approach performed in different times during the fattening cycle provides important information on animal health and welfare and could help farmers improve welfare in commercial turkey flocks.

## Figures and Tables

**Table 1:** Information relating to flocks of turkeys: number of birds and hybrids, age of birds at each assessment, veterinary treatment, age, and average live weight at slaughter.

Flock	Barn Size (m^2^)	Number of Birds Placed		Hybrid	Age of Birds at Each Assessment (Days)			Veterinary Intervention	Age at Slaughter (Days)		Average Live Weight (kg)	
		Toms	Hens		I	II	III		Toms	Hens	Toms	Hens
1 ^1^	1100	3900	3700	Converter	22	91	132	Yes ^3^	143	97	20.6	9.25
2 ^2^	1100	3900	3700	Converter	22	91	132	Yes ^3^	143	97	20.4	9.23
3	900	2300	2000	Converter	21	84	125	Yes ^4^	143	98	20.28	9.47
4	1380	4000	3300	BUT 6	22	85	126	Yes ^3^	147	102	20.36	8.98
5	960	2700	2200	BUT 6	28	92	135	Yes ^3^	147	104	19.67	9.28
6	1440	3400	3300	BUT 6	27	91	131	Yes ^3^	150	101	19.27	9.19

^1^ At the age of 38 days, a total of 1305 males and 1245 females were taken out and placed on another farm. ^2^ At the age of 39 days, a total of 1260 males and 1170 females were taken out and placed on another farm. ^3^ Birds were treated because of necrotic enteritis at 5 to 6 weeks of age. ^4^ Birds were treated due to *E. coli* infection at 1 and 5 weeks of age.

**Table 2:** Number of birds examined, cumulative mortality, and stocking density at each assessment.

Flock	Sex	First Assessment			Second Assessment			Third Assessment		
		Birds Exam.	Mortality (%)	Stock. Density (Birds/m^2^)	Birds Exam.	Mortality (%)	Stock. Density (Birds/m^2^)	Birds Exam.	Mortality (%)	Stock. Density(Birds/m^2^)
1	M	3818	2.11	5.78	2435	3.73	3.85	2360	7.66	1.86
	F	3543	0.95	8.32	2390	1.81	5.43			
2	M	3844	1.44	5.82	2430	2.66	3.85	2335	5.49	2.26
	F	3665	1.25	8.08	2344	1.77	5.35			
3	M	2221	3.43	8.23	2168	5.74	4.01	2088	8.21	2.32
	F	1936	3.20	10.76	1903	4.85	5.28			
4	M	3956	1.10	9.15	3846	3.85	4.45	3790	5.25	2.63
	F	3262	1.15	11.32	3242	1.75	5.62			
5	M	2653	1.74	9.21	2556	5.33	3.90	2481	8.11	2.58
	F	2173	1.23	11.31	2149	2.32	5.59			
6	M	3270	3.82	5.66	3203	5.79	4.67	3168	6.82	2.77
	F	3195	3.18	8.31	3166	4.06	6.94			

**Table 3:** Description of the birds’ behavior and appearance in each of the welfare indicator categories.

**Indicator**	**Description**
Immobile	Birds not moving when approached or, after being gently touched, only able to move by propping themselves up on their wings
Lame	Birds walking with obvious difficulties; one of the legs not placed on the ground, bird moving away from the observer but stopping after two to three paces to rest
Head wounds	Visible alterations on the head, snood, beak, or neck related to fresh or older wounds
Back wounds	Bird with visible fresh or older wounds on the back, wings, or legs
Vent wounds	Visible wounds around tail, or on its sides, including fresh, older, or bleeding wounds
Small size	Birds that are approximately 50% the size of an average bird in the flock
Featherless	Missing or damaged feathers on the majority of the back area, including the wings and tail
Dirty	Very clear and dark staining of the back, wing, and/or tail feathers of the bird, covering at least 50% of the body area
Sick	Bird showing mild to severe clinical signs of impaired health; pale comb and eyes, watery discharge, and swollen sinuses, visibly breathing
Dead	Dead birds found during the assessment
Pendulous crop	Birds with a pendulous crop hanging in front of the breast
Aggression toward birds	Bird chases or pecks, hits, flies into, or leaps onto another bird
Aggression toward humans	Bird perceptibly hits human with the wings, or runs into, jumps onto, or pecks the human

**Table 4:** Welfare indicator mean values for male and female turkeys expressed as percentage at each assessment.

		Assessment						
		I (3–4 w)			II (13–14 w)			III (17–19 w)
Indicator	Sex	*n (%)*	*z*	*p*-Value	*n (%)*	*z*	*p*-Value	*n* (%)
Immobile	*M*	15 (0.076)	−0.06	ns	62 (0.372)	3.16	0.002 ***	86 (0.530)
	*F*	14 (0.078)			28 (0.184)			
Lame	*M*	3 (0.015)	−0.14	ns	11 (0.066)	−0.83	ns	32 (0.197)
	*F*	3 (0.017)			14 (0.092)			
Head wounds	*M*	2 (0.010)	−0.09	ns	8 (0.048)	−1.10	ns	2 (0.012)
	*F*	2 (0.011)			12 (0.079)			
Back wounds	*M*	4 (0.020)	−0.12	ns	12 (0.072)	1.00	ns	5 (0.031)
	*F*	4 (0.022)			11 (0.072)			
Vent wounds	*M*	3 (0.015)	−0.14	ns	11 (0.066)	1.65	0.100 *	0 (0)
	*F*	3 (0.017)			4 (0.026)			
Small size	*M*	197 (0.997)	2.66	0.008 ***	21 (0.126)	1.09	ns	21 (0.129)
	*F*	128 (0.721)			13 (0.086)			
Featherless	*M*	6 (0.030)	0.75	ns	164 (0.986)	−1.38	ns	49 (0.302)
	*F*	3 (0.017)			174 (1.145)			
Dirty	*M*	4 (0.020)	1.74	0.081 *	343 (2.062)	6.00	0.001 ***	86 (0.530)
	*F*	0(0)			183 (1.204)			
Sick	*M*	6 (0.030)	2.14	0.033 **	4 (0.024)	−1.98	0.048 **	17 (0.105)
	*F*	0 (0)			11 (0.072)			
Dead	*M*	0 (0)			2 (0.012)	1.35	ns	12 (0.074)
	*F*	0 (0)			0(0)			
Pendulous crop	*M*	0 (0)			10 (0.060)	−1.04	ns	9 (0.055)
	*F*	0 (0)			14 (0.092)			
Agression towards birds	*M*	0 (0)			0 (0)			2 (0.012)
	*F*	0 (0)			0 (0)			
Agression towards humans	*M*	0 (0)			0 (0)			2 (0.012)
	*F*	0 (0)			0 (0)			

**Note:** Population of birds examined in each visit: first N**_male_** = 19,762, N**_female_** = 17,754; second N**_male_** = 16,638, N**_female_** = 15,194; third N**_male_** = 16,222. Significance: * *p* < 0.1; ** *p* < 0.05; *** *p* < 0.01.

**Table 5:** Mean temperature and CO_2_ and NH_3_ values in turkey facilities at each assessment.

Parameter	Flock					
	1	2	3	4	5	6
***Assessment I***						
Inside temp. (°C)	26.5	26.6	26.7	28.9	26.8	27.0
NH_3_ (ppm)	40	27.6	4	0	0	0
CO_2_ (ppm)	4433	3000	1630	1200	1050	975
***Assessment II***						
Inside temp. (°C)	21.5	21	19.5	26.0	26.5	27.6
NH_3_ (ppm)	3	2.3	4	0	0	0
CO_2_ (ppm)	1600	1217	1750	1200	950	1025
***Assessment III***						
Inside temp. (°C)	25	28	23.2	21	23	20.5
NH_3_ (ppm)	0	0	0	0	2.3	0
CO_2_ (ppm)	1000	850	867	1000	2200	1000

**Note**: Recommended values according to Aviagen: ambient temperature at 3–4 weeks = 23–25 °C, at more than 9 weeks = 16–17 °C; NH_3_: <20 ppm; CO_2_: <2500 ppm [27].

**Table 6:** Welfare indicators’ prevalence (including mortality) by different CO_2_ and NH_3_ levels.

Parameter	Observations *n* (%)	Prevalence *n* (%)	Odds Ratio (CI)	SE	*p*-Value
CO					
<1600 ppm	247 (63.33)	106 (42.91)	1		
1600–3000 ppm	91 (23.33)	32 (35.16)	0.20 (0.07–0.54)	0.103	0.002 ***
>3000 ppm	52 (13.33)	10 (19.23)	0.15 (0.04–0.55)	0.100	0.004 ***
NH3					
0 ppm	221 (56.67)	86 (38.91)	1		
>0 ppm	169 (43.33)	62 (36.69)	0.35 (0.12–1.02)	0.192	0.056 *

Note: * Significance at *p* < 0.1; *** *p* < 0.01.

## Data Availability

Not applicable.
